# Potential ‘Ecological Traps’ of Restored Landscapes: Koalas *Phascolarctos cinereus* Re-Occupy a Rehabilitated Mine Site

**DOI:** 10.1371/journal.pone.0080469

**Published:** 2013-11-25

**Authors:** Romane H. Cristescu, Peter B. Banks, Frank N. Carrick, Céline Frère

**Affiliations:** 1 School of Biological, Earth and Environmental Sciences, University of New South Wales, Kensington NSW, Australia; 2 Centre for Mined Land Rehabilitation, Sustainable Minerals Institute, The University of Queensland, St Lucia QLD, Australia; 3 School of Biological Sciences, The University of Sydney, Camperdown, Sydney NSW, Australia; 4 Centre for Ecology and Conservation, The University of Exeter, Penryn, Cornwall, United Kingdom; Macquarie University, Australia

## Abstract

With progressively increasing anthropogenic habitat disturbances, restoration of impacted landscapes is becoming a critical element of biodiversity conservation. Evaluation of success in restoration ecology rarely includes faunal components, usually only encompassing abiotic and floral components of the ecosystems. Even when fauna is explicitly included, it is usually only species presence/absence criteria that are considered. If restoration is to have a positive outcome, however, populations in restored habitats should exhibit comparable survival and reproductive rates to populations found in undisturbed surroundings. If a species recolonises restored areas but later experiences decreased fitness, restored areas could become ecological sinks or traps. We investigated this possibility in a case study of koalas *Phascolarctos cinereus* occupying rehabilitated mining areas on North Stradbroke Island, Australia. Our holistic approach compared rehabilitated and undisturbed areas on the basis of their vegetation characteristics, of koalas' body condition, roosting trees, diet, as well as predator index. Koalas using rehabilitated areas appeared to be able to access an adequate supply of roosting and fodder trees, were in good condition and had high reproductive output. We did not find any significant differences in predator density between rehabilitated areas and undisturbed surroundings. The results presented in this study showed there was no evidence that the post-mining rehabilitated areas constitute ecological sinks or traps. However, to reach a definitive conclusion as to whether areas rehabilitated post-mining provide at least equivalent habitat to undisturbed locations, additional research could be undertaken to assess foliar nutrient/water/toxin differences and predation risk in rehabilitated areas compared with undisturbed areas. More generally, the evaluation of whether restoration successfully produces a functional ecological community should include criteria on the fitness of faunal populations reoccupying such sites, so as to ensure functioning ecosystems, rather than ecological sinks or traps, are the outcome.

## Introduction

Globally, pristine environments available for protection are decreasing, while degraded environments in need of restoration are increasing [1]. While it would clearly be preferable to avoid impacts on remaining undisturbed areas [2], the demand for resources conflicts with this approach. As a result, restoration of degraded land now plays an increasingly crucial role in the conservation of biodiversity [3].

Restoration of disturbed habitats has the ultimate goal of supporting self-sustaining assemblages that characterised the habitat prior to disturbance [4]. Commonly, restoration success is assessed on abiotic and selected biotic (i.e. flora) elements. Faunal elements are more rarely incorporated into monitoring of rehabilitation success and when they are, mostly there is reliance on presence/absence data only. However, even if a species does recolonise an impacted site, restoration for that species can only be regarded as a success if its population is stable in the long-term and shows levels of fitness equal to those found in pre-disturbance habitats. That is, rehabilitated areas should not be population sinks [5], [6] nor should they function as ecological traps. An ecological trap arises when a species expresses a maladaptive choice: for example, when an animal settles in a habitat where its fitness is decreased relative to what it could be in other available habitats [7]. To identify such processes, the success of restoration should not only be measured in terms of the return of communities, but also should ultimately be measured in terms of factors that reflect the re-establishment of populations with demographic patterns that are similar to those of undisturbed populations.

Habitat restoration associated with post-mine rehabilitation is rapidly expanding and improving, driven, in some places, by the impetus of a legislative framework (e.g. in USA with the U.S. Surface Mine Control and Reclamation Act of 1977), public concern and the industry itself [8], [9], [10]. Post-mine rehabilitation provides an ideal framework to study the fate of fauna populations in restoration projects, as it is readily available and its legislative requirement enables the monitoring of rehabilitated areas to achieve regulatory standards [11].

This study investigated and compared some ecological characteristics in rehabilitated post-mine landscapes and nearby undisturbed landscapes on North Stradbroke Island (NSI), Australia, using the koala, *Phascolarctos cinereus*, as a model. The koala, an arboreal folivorous marsupial, was classified in 2012 as a nationally vulnerable species in Australia under the Commonwealth Government's *Environment Protection and Biodiversity Conservation Act 1999*. As a charismatic large mammal its persistence and welfare is important to all stakeholders. In light of decreasing koala populations due to habitat loss [12], testing the ability of restoration to recreate viable habitat for koalas is critical. On NSI, a sand mining company is progressively rehabilitating its mine path with more than 3000ha currently available for fauna recolonisation. In order to test predictions from the theories of population sinks and ecological traps, we hypothesised the following possible scenarios.

i) A population sink could occur if the rehabilitated habitat is of inferior quality. Individuals would not show a preference to settle in the sink but would overflow from high quality habitats and animals inhabiting rehabilitated habitats would have lower survival and reproductive rates [5]. A lower survival rate could result from a higher predator density in rehabilitated areas. Indeed, some studies have found an association between disturbances and feral species, including feral predators such as European foxes *Vulpes vulpes* and feral dogs *Canis lupus* [13], [14]. Although adult koalas are probably not vulnerable to fox attacks [though anecdotal accounts of fox attacks on adults have been reported, 15], juvenile koalas fall within the fox prey-size range [16] and feral dogs can attack koalas of any size [14]; indeed dog attacks are one factor driving koala populations toward extinction [17]. In addition, small trees in younger rehabilitated areas may not provide sufficient shelter from aerial predators such as eagles and owls [18], [19], or may require increased movements between trees (and thus greater vulnerability to terrestrial predators) owing to lower leaf mass per tree for feeding. Lower reproductive rates could be a result of lower food quantity and/or quality, as well as an increased energy expenditure associated with movements to find appropriate food trees.

ii) In ecological traps, contrary to sinks, animals choose to settle in lower-quality habitat despite high-quality habitat being available. An equal-preference trap can occur in rehabilitated habitat if individuals do not discriminate between rehabilitated and undisturbed habitats, but these two habitats have different suitability [7]. This could happen if rehabilitated habitats present the same cues of suitability as undisturbed habitat but produce lower survival or reproductive rates - for the same potential reasons as i). A worse kind of ecological trap that could potentially occur for koalas on NSI is the severe trap, where animals favour the less suitable habitat instead of more suitable habitats [7]. A severe trap could be created if rehabilitated areas are more attractive to koalas than undisturbed areas, while reproduction or survival rates are decreased. Young trees in rehabilitated areas may be more attractive than surrounding undisturbed habitat as koalas favour leaves that have a high concentration of crude protein and lower fibre content [20], [21], which are typical of fast growing trees [22].

On the basis of these population source/sink and ecological trap hypotheses, we can make some general predictions: i) if the rehabilitation area functions as a population sink, we predict that the habitat there would be of low quality (*e.g.* low tree density and species richness, particularly for specific koala food trees, high predator density), that the koalas residing there would be at low density, have poor general condition and low reproductive rate; ii) if the rehabilitation area functions as an ecological trap, we predict that although the habitat quality may be low as in the sink scenario above, the density of resident koalas would be equal to or higher than the density of those residing in undisturbed areas (as koalas would not avoid the rehabilitated area), but that koalas would present the same signs of low survival and reproductive rate as in the sink hypothesis; and finally iii) if the rehabilitated area provides good quality habitat, we predict that the habitat would present high levels of koala tree density and species richness, with resident koalas presenting indicators of survival and reproductive rate comparable to those in undisturbed areas, *i.e.* equal or lower predator density, equal or higher koala density and individuals exhibiting good body condition and breeding success.

The consequences of restoration projects which inadvertently create ecological sinks or traps could be devastating, so their potential occurrence should be investigated [5], [7]. Accordingly, our study aimed to consider the above hypotheses by investigating different ecological components relevant to the habitat selection theories of sources/sinks and traps for koalas in rehabilitated areas. We compared, in undisturbed and rehabilitated koala habitats, key elements of habitat quality such as vegetation composition and structural characteristics, roosting tree availability, and the presence of feral predators; as well as providing information on koala diet, health and reproduction. The study succeeded in demonstrating that these comparisons did not support hypotheses i) or ii) and thus indicated the post-mining restored landscapes provided good quality habitat for koalas.

## Materials and Methods

### Study site and characteristics

North Stradbroke Island is a sand island located in Moreton Bay (27°34′S, 153°28′E) off the coast of south-eastern Queensland, Australia. It is predominantly formed of unconsolidated Cainozoic sediments [23], with a wet-dry subtropical climate [24]. Sand mining has been conducted on the island since the late 1940s to extract heavy minerals from dredged sand. The mined area is progressively rehabilitated: dune landform is recreated, topsoil is spread and stabilised, then seeds and tube-stock trees are planted out. Previously, it is likely that mining activity has been undertaken in koala habitat, with these areas now being in various stages of rehabilitation. Preliminary surveys in undisturbed areas contiguous with those rehabilitated areas have confirmed the presence of koalas (RC, *unpublished data*). Thus remnant koala populations exist in proximity to rehabilitated areas and enable recruitment.

### Koala habitat characteristics

The landscape was surveyed for koala faecal pellets or scats using plots. These plots were set on transects distributed to sample rehabilitated vegetation established in different years. In areas representative of each different year of rehabilitation, transects were chosen randomly, but run across the landscape. The first plot was randomly placed along the transect then plots were spread 200 m apart to achieve one plot for 6ha. Plots (50×10 m, N = 36) containing koala scats were selected to compare vegetation characteristics in rehabilitated and undisturbed koala habitats. Plot coordinates were recorded by hand held GPS (Garmin, eTrex®H, USA, accuracy ±7 m) using UTM in AMG 84 projection. The number of koala scats per plot was recorded, as an index of intensity of use. Previous experiments found variation in scat detectability and decay rates between different habitats on the island [25]. Thus the scat count was multiplied by a correction factor for the lower detectability characteristic of undisturbed plots (correction factor = 1.2 in complex litter [25]); no correction for higher scat decay rate was necessary as no plot was located in zones subject to flooding. A single researcher (RC) conducted all surveys to eliminate bias resulting from heterogeneity in observer skills [26].

The vegetation characteristics of koala habitat in undisturbed areas were assessed by sampling all remnant vegetation communities surrounding the mine. These remnants indicated the vegetation communities most likely to have been present before mining, which were subsequently replaced by rehabilitated areas. Vegetation communities were based on Regional Ecosystem (RE) maps [27], with twelve plots in four vegetation communities selected (Table 1).

**Table 1:** Regional Ecosystems (RE) potentially replaced by mining rehabilitated areas and description of their floristics.

RE	community
12.2.6	*Eucalyptus racemosa*, *Corymbia intermedia*, *C. gummifera*, *Angophora leiocarpa* and *E. pilularis* shrubby or grassy woodland to open-forest
12.2.7	*Melaleuca quinquenervia* open-forest to woodland with *E. tereticornis*, *C. intermedia*, *E. robusta*, *Lophostemon sp*;
12.2.8	*E. pilularis* and *E. resinifera* open-forest
12.2.10	Mallee forms of *C. gummifera*, *E. racemosa* and *E. planchoniana* ± *Banksia aemula* low shrubby woodland

Rehabilitated areas were divided into three categories based on changes in rehabilitation methodology. Prior to 1987, rehabilitation was designed to stabilise landforms and involved exotic as well as native plant species (6 plots). After 1987, a new rehabilitation policy was developed in which only native species were used and the use of *Acacia sp*. (black wattle) ceased, as it had been found that the acacias were outcompeting other species such as eucalypts (10 plots). After 1998, the method once again was refined: the previous extensive use of *Allocasuarina sp.* (a species that was reaching excessive densities in rehabilitated areas) ceased. Moreover, only seeds collected on the island were used (8 plots). The improvements in methodology (including increases in the number of native species used, of tube stock plantings, the translocation of flora species of interest, etc.) were deliberate attempts to improve the habitat quality of the rehabilitated areas. The improvements of methodology were based on benchmarking [28], i.e. the comparisons of the efficiency of other methodologies used in the industry as well as the results of local research on rehabilitation success (Ben Barker, *personal communication*, 28/09/2010).

In the 36 plots described above, environmental and vegetation variables were recorded using the methodology developed for the mine's environmental monitoring program, or using the mining database, both of which are based on Queensland Herbarium recommended methodology (Sibelco, *unpublished data*). Native tree species richness and density were calculated, with a tree being defined as any live stem greater than 2 m high. Each tree was marked to ensure it was only counted once. Density and species richness for the main koala food trees of the genera *Eucalyptus* and *Corymbia* (E+C) were extracted [29]. For these genera, the circumference at breast height (CBHp for circumference of trees in plots) was measured. When multiple stems occurred, all CBHp were added. The percentages of canopy and ground cover were estimated every two meters along the two transects forming the longer borders of the plots [30]. Elevation, slope and aspect of the plots were extracted by Terramodel Version 10.61 from a 2008 airborne laser scan of the island (Sibelco, *unpublished data*).

### Study animal characteristics

This study was carried out under the Queensland Environmental Protection Agency wildlife permits (WISP00491302 and WITK05609808) and The University of Queensland animal ethics approval (project permit ID 206/07 and 314/08). Eight koalas (six females, two males) were caught according to standard procedures [31]; seven of these were captured near rehabilitated areas and the eighth was captured inside one. Tooth-wear classes [32] and body condition [33] were assessed, and blood samples were collected (5 ml, cephalic vein). Reproductive status was determined for females by pouch checks.

### Movement patterns and habitat use

Koalas were fitted with radio-tracking collars, each transmitter being set to a different VHF frequency between 150–152 MHz (Titley Electronics, Australia) and released back into the tree where they had been captured. Collared koalas were radio-tracked every one or two weeks from July 2008 to February 2010. Their location was recorded by hand held GPS. The positions were plotted on the map of vegetation communities of the island [27] superimposed with contours of rehabilitated areas by year (Sibelco, *unpublished data*). Home ranges were plotted using the Home Range Tools for ArcGIS® 1.1. [34]. We used the kernel density estimation method, with the standard Gaussian curve [35]. Based on the Schoener index [36], the variances of our coordinates were unequal so the data were standardised. We used a fixed kernel [37], with a smoothing factor calculated by least squares cross validation [38]. Home range areas were calculated from isopleths of the volume contours. The percentages of each vegetation community inside the 95% isopleth of the home ranges were extracted in ArcGIS 9.3.1.

### Roosting trees

Each tree where a collared koala was found was classified as belonging to undisturbed or rehabilitated area. Trees were tagged with a unique number to allow recording of reuse by the same or other koalas. Roosting tree species and circumference at breast height of trees used by koalas (CBHk) were recorded.

### Diet

Koala diet was evaluated by identification of the cuticle characteristics of leaf fragments remaining in the scats [39], [40], [41]. While many studies have used scats to determine diets, this method is not without problems, differential digestibility being the most serious. However in feeding experiments in koalas, the proportion of different fodder species fed to koalas was reflected by the proportion of fragments found in scat analysis and different fodder species had the same gut transit time [40].

When a collared koala was located, the area directly under it and the base of its tree were searched for fresh scats (i.e. covered in mucous and smelling of eucalypts oil). Only fresh scats (less than a day or two old) were collected, to ensure the scats belonged only to the radio-tracked koala and reflected browse usage of the season in which they were collected.

For each individual koala included in the diet analysis (N = 5), we selected four groups of scats produced when the koala was in rehabilitated areas and an additional four groups of scats produced when the koala was in undisturbed areas. We selected the groups of scats as equally as possible across seasons (Summer = 11, Autumn = 6, Winter = 11, Spring = 12). Each group of scats represented five scats collected during the same occasion and homogenised for analysis of leaf fragments [39], [41].

A NSI leaf library was prepared to assist in dietary scat analysis. Every tree species found during the study was sampled (on average 20 leaves per sample), including species not typically recognised as koala food, to avoid bias from preconceptions. Where possible, tree species were sampled four times: in rehabilitated and undisturbed areas at two separate geographic locations. The precise characteristics of leaf cuticle, for stomata in particular, were described and compared.

Reference specimens from the leaf library enabled the identification of 100 leaf fragments extracted from each koala scat group. This procedure was repeated twice and the percentage of tree species present in each scat group was calculated.

### Predator index

An Activity Index [42] was calculated for foxes and feral dogs. Sand was flattened and smoothed in 2×2 m plots, 1 km apart, along tracks at the study sites at dusk. Plots in undisturbed areas (N = 16) and in rehabilitated areas (N = 14) were monitored at dawn on three consecutive days over three survey periods (October 2003, December 2005 and November 2009). Plots that were unreadable owing to weather conditions (e.g. heavy rain) or tyre tracks were excluded from calculations using the criteria of the Activity Index method.

### Data analysis

All variables were tested for normality and homogeneity of variances (Levene's test of homoscedasticity) and were compared between the three groups of rehabilitated areas and the undisturbed area by appropriate parametric or non-parametric tests in PASW Statistics 18.0 [43]. Significance level was taken to be p<0.05 (except when accounting for Bonferroni's adjustment), effect size [as defined in 44], standard deviation (SD) or standard error of mean (SEM) being calculated when appropriate [45].

Similarity and dissimilarity matrices were constructed using the Bray-Curtis measure [46] on the plant species in the plots. Square root transformation was used to down-weight the importance of abundant species. Differences between the three rehabilitated areas and undisturbed area were tested with analyses of similarities [ANOSIM, 47], a multivariate equivalent of the analysis of variance (ANOVA) based on similarity matrices [48]. Matrices were also constructed for browse species found in koala scats. Browse species were compared between koalas, for different seasons and locations (rehabilitated/undisturbed areas) with ANOSIM and non-parametric multidimensional scaling (nMDS, between locations only) based on a squared transformed Bray-Curtis matrix. These analyses were performed using Primer 6.1.12 [49].

## Results

### Recolonisation pattern

Signs of use by koalas were found in rehabilitated areas as young as 6 years post rehabilitation (the youngest age checked) and in plots representing all rehabilitation methods. The number of scats per plot ranged from 1 to 444. The average number of scats, corrected for variation of scat detectability, was similar in each rehabilitated habitat and undisturbed habitat (undisturbed habitats: 23.8 SEM = 7.8, pre-87 habitats: 20.8 SEM = 6.9; 88–97 habitats: 104.5 SEM = 43.2; post-98 habitats: 70.9 SEM = 49.8; Kruskal-Wallis test = 2.27, df = 3, p = 0.518).

### Koala habitat characteristics

The differences of vegetation characteristics between rehabilitated and undisturbed habitats are presented in detail in Table 2 (see also Supplementary Information Figure S1). In rehabilitated compared to undisturbed habitats, tree density and richness were greater, E+C (i.e. *Eucalyptus* and *Corymbia*) density and richness were either equal or greater, percentages of E+C were either equal or greater and trees were smaller (even in 31-year-old rehabilitated habitats). Canopy cover and proportion of bare ground were similar for all habitats rehabilitated before 1997 and undisturbed habitats, while habitat rehabilitated after 1998 had less canopy and more bare ground. Elevation, aspect and slope were similar between the three rehabilitated habitats and undisturbed habitats (Kruskal-Wallis tests respectively: 3.51, df = 3, p = 0.319; 1.95, df = 3, p = 0.583; 4.62, df = 3, p = 0.201).

**Table 2:** Characteristics of vegetation composition and structure in rehabilitated (R) koala habitats classified by method compared to undisturbed (U) koala habitats.

	rehabilitated habitats (R)						U			effect size	R compared to U	p	test statistics	df	test
	pre-87		88 to 97		post-98										
	mean	*SEM*	mean	*SEM*	mean	*SEM*	mean	*SEM*							
**Density of trees**	3617	*1315*	1572	*376*	1513	*406*	902	*145*		1.5	superior	0.017	10.18	3	Kruskal-Wallis
**Density of E+C**	310	*85*	501	*144*	884	*337*	207	*69*		1.7	equal	0.059	2.75	35	ANOVA
**Richness of trees**	7.5	*0.5*	10.0	*0.5*	9.6	*0.3*	5.3	*0.5*		0.7	superior	<0.001	24.15	35	ANOVA
**Richness of E+C**	3.0	*0.4*	5.1	*0.4*	5.1	*0.4*	1.9	*0.3*		1.3	superior	<0.001	22.37	35	ANOVA
**Percentage E+C**	0.22	*0.05*	0.62	*0.07*	0.73	*0.07*	0.26	*0.06*	pre-87	−0.1	equal	0.892	34.00		Mann-Whitney U
									88 to 97	1.4	superior	0.002	15.00		Mann-Whitney U
									post-98	1.8	superior	0.001	4.00		Mann-Whitney U
**CBHp**	45	*8*	27	*3*	19	*2*	114	*15*		−0.7	inferior	<0.001	19.16	35	ANOVA
**Canopy cover %**	83	*3*	69	*5*	37	*5*	74	*5*	pre-87	0.1	equal	0.265	1.16	16	T test
									88 to 97	−0.1	equal	0.505	−0.68	20	T test
									post-98	−0.5	inferior	<0.001	−4.67	18	T test
**Bare ground %**	3	*1*	4	*1*	32	*4*	4	*1*	pre-87	−0.1	equal	0.453	28.00		Mann-Whitney U
									88 to 97	0.0	equal	0.620	52.50		Mann-Whitney U
									post-98	7.9	superior	<0.001	0.00		Mann-Whitney U

There was a significant difference in tree species composition (n.b. all trees in this analysis are species used by koalas as roosting and/or food trees) in the three rehabilitated habitat groups and undisturbed habitats (ANOSIM, R = 0.391, p<0.001). The undisturbed plots, which comprised different RE vegetation communities (Table 1), were less similar to one another than were the rehabilitated plots. Habitats rehabilitated using different methods were more similar to each other than to undisturbed habitats (Table 3). However, the dissimilarity between rehabilitated and undisturbed habitats decreased with the change of rehabilitation methods, with the more recent methods producing vegetation associations more similar to adjacent undisturbed areas. The main tree species contributing to the similarities were *Corymbia sp*. and *Banksia sp*. in undisturbed areas, and *Allocasuarina sp*. and *Callitris sp*. in pre-97 rehabilitated areas, whereas similarities in post-98 rehabilitated areas were driven by *Allocasuarina sp*., *Corymbia sp*. and *E. pilularis* (other details in Table 3).

**Table 3:** Average tree species composition similarity inside a group and dissimilarity between groups for rehabilitated and undisturbed koala habitats. The tree species given are explaining 50% of similarity/dissimilarity.

Areas	undisturbed	rehabilitated		
		pre-87	88 to 97	post-98
**Undisturbed**	**42.4**			
	*Banksia sp + Corymbia sp*			
**Rehabilitated pre-87**	**73.8**	**54.8**		
	*Allocasuarina sp + Callitris sp + Banksia sp*	*Allocasuarina sp + Callitris sp*		
**Rehabilitated 88 to 97**	**69.2**	**46.0**	**61.4**	
	*Allocasuarina sp + Banksia sp + Callitris sp*	*Callitris sp + Allocasuarina sp + E. racemosa*	*Allocasuarina sp + Callitris sp*	
**Rehabilitated post-98**	**67.5**	**53.7**	**42.5**	**60.7**
	*Banksia sp + Allocasuarina sp + Corymbia sp + E. planchoniana*	*Callitris sp + Allocasuarina sp + Corymbia sp*	*Allocasuarina sp + Corymbia sp + Callitris sp + E. racemosa*	*Allocasuarina sp+ Corymbia sp + E. pilularis*

### Study animal characteristics

Based on tooth wear, captured koalas were between 2 and 14 years of age [32]. All koalas, but one, were in good body condition (condition index scores of 7 to 9), the body condition of the other animal caught was lower. Blood analysis indicated that haematology and biochemistry values were in normal ranges [50], with some koalas having minor changes of no biological significance. Out of the six females caught, five had pouch or back young, the other was immature (<3 years).

### Habitats used

**Figure 1:**
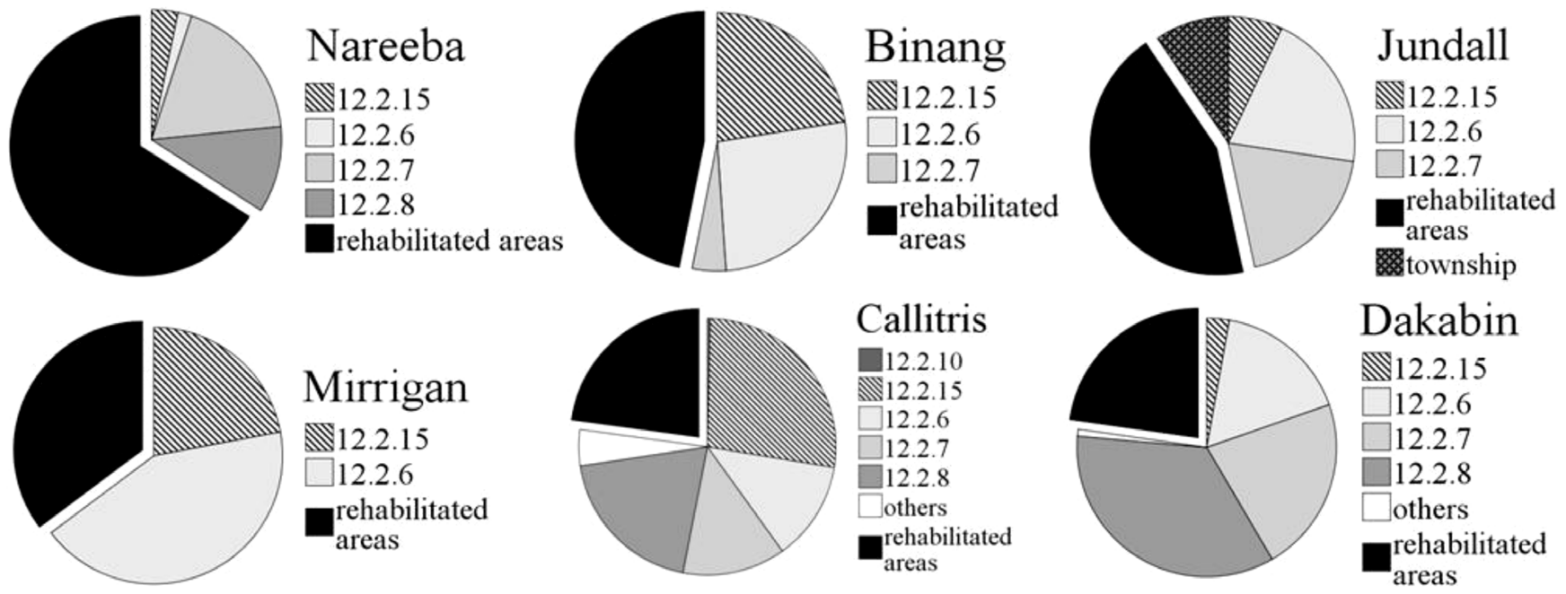
Percentages of rehabilitated areas (in decreasing order) in the home ranges of six koalas: Nareeba (65.9%), Binang (46.9%), Jundall (43.9%), Mirrigan (35.3%), Callitris (22.8%) and Dakabin (22.7%); together with the different remnant vegetation communities present in their home ranges (based on REs, RE12.2.15: wetlands, other REs: see **Table 1**).

Six out of seven koalas caught near rehabilitated areas were subsequently found in those areas. The signal from the last koala was lost, apparently due to VHF transmitter failure. The eighth koala was captured and subsequently found only in rehabilitated areas. Added home ranges (95% kernel) for all koalas were mainly composed of rehabilitated areas (44.5% SD = 18.7), followed by *Eucalyptus* and *Corymbia* woodland RE12.2.6 (17.5% SD = 9.4), *Melaleuca* woodland RE12.2.7 (13.5% SD = 8.9), wetlands RE12.2.15 (12.2% SD = 3.6), and *Eucalyptus* open-forest RE12.2.8 (7.6% SD = 3.5). The details for each koala are presented in Figure 1.

### Roosting trees

Individual collared koalas were found in trees inside rehabilitated areas for 23% to 100% (mean = 51%, SD = 26%) of observations. Results for the seven koalas using rehabilitated areas were pooled to calculate the following results, except as otherwise stated. There were 258 observations of koalas in identified roosting trees: 109 in undisturbed areas and 149 in rehabilitated areas (Table 4). Roosting tree species differed between undisturbed and the three rehabilitation groups (χ^2^ = 144.37, df = 48, p<0.001, contingency coefficient = 0.604, p<0.001). In pair-wise comparisons, the most significant differences in roosting trees were between undisturbed areas and pre-1987 rehabilitated areas; then between pre-1987 and post-1998 rehabilitated areas (Table 5, Bonferroni's adjustment α = 0.008).

**Table 4:** Number of times each tree species and each unique tree were used or reused in undisturbed and rehabilitated areas by radio-tracked koalas (N = 7).

	number of times each species has been used						number of times reused (number of reused trees)	
	undisturbed			rehabilitated			undisturbed	rehabilitated
Tree species	rank	number	%	rank	number	%		
*E. robusta*	1	27	24.8	3	16	10.7	7 (3)	13 (4)
*E. racemosa*	2	15	13.8	2	23	15.4		4 (2)
*Banksia sp*	3	13	11.9	7	5	3.4	2 (1)	
*Lophostemon sp*	3	13	11.9	9	2	1.3	2 (1)	
*Callitris sp*	4	11	10.1	1	47	31.5	4 (2)	
*E. tereticornis*	5	6	5.5					
*Corymbia sp*	6	5	4.6	8	4	2.7		
*E. pilularis*	6	5	4.6	5	12	8.1		
*Allocasuarina*	7	4	3.7	4	13	8.7		
*Angophora sp*	7	4	3.7	10	1	0.7		
*Acacia sp*	8	2	1.8					
*Melaleuca sp*	8	2	1.8	6	11	7.4		
*Duboisia sp*	9	1	0.9					
*Schefflera sp (umbrella tree)*	9	1	0.9					
*E. planchoniana*				9	2	1.3		
*E. resinifera*				6	11	7.4		2 (1)
*E. tindaliae*				9	2	1.3		
**TOTAL**		**109**			**149**		**15 (7)**	**19 (7)**
**PERCENTAGE**		**42.2%**			**57.8%**		**13.7% (7%)**	**12.7% (5.1%)**

**Table 5:** Significance level of the difference in roosting trees used by radio-tracked koalas (N = 7) in rehabilitated and undisturbed areas (Mann-Whitney U tests).

Areas		undisturbed		rehabilitated			
				pre-87		88 to 97	
		test statistics	p values	test statistics	p values	test statistic	p value
**Rehabilitated**	**pre-87**	2704.5	0.001				
	**88 to 97**	2473.5	0.003	1767.5	0.450		
	**post-98**	505.5	0.809	133.0	0.005	192.0	0.199

No difference was found between roosting trees in areas rehabilitated after 1998 and undisturbed areas (these two groups were also the most similar in tree species composition, as indicated previously). At the individual animal level, out of the six koalas using both rehabilitated and undisturbed areas, five were using different species of roosting trees in each (Table 6), while the last individual used only areas rehabilitated after 1998, which were the most similar to undisturbed areas in terms of tree species composition.

**Table 6:** Significance of the results of similarity between roosting tree species used in rehabilitated and undisturbed habitats, for each of six koalas using both habitats.

	?^2^	df	p
**Binang**	21.6	8	0.006
**Callitris**	31.9	11	0.001
**Dakabin**	6.1	7	0.525
**Jundall**	24.2	11	0.012
**Mirrigan**	18.8	9	0.027
**Nareeba**	15.6	6	0.016

Koalas used 14 roosting tree species in undisturbed areas and 13 in rehabilitated areas, with 10 species common to both (Table 4). The roosting species most often used in undisturbed areas was *E. robusta* (25%), whilst *Callitris sp*. was the most commonly used in rehabilitated areas (32%). Two of the three most frequently used trees were the same in undisturbed and rehabilitated areas (*E. robusta* and *E. racemosa*). The number of roosting trees that koalas used multiple times was quite low and similar (χ^2^ = 0.567, df = 3, p = 0.903) in undisturbed (7%) and rehabilitated areas (5.1%). Mean CBHk of the trees used in undisturbed areas (138 cm, SEM = 9.6) was unsurprisingly larger than in rehabilitated areas (89 cm, SEM = 5.7, Mann-Whitney U test = 4999, p<0. 001), reflecting the size of the trees available.

### Diet

**Figure 2:**
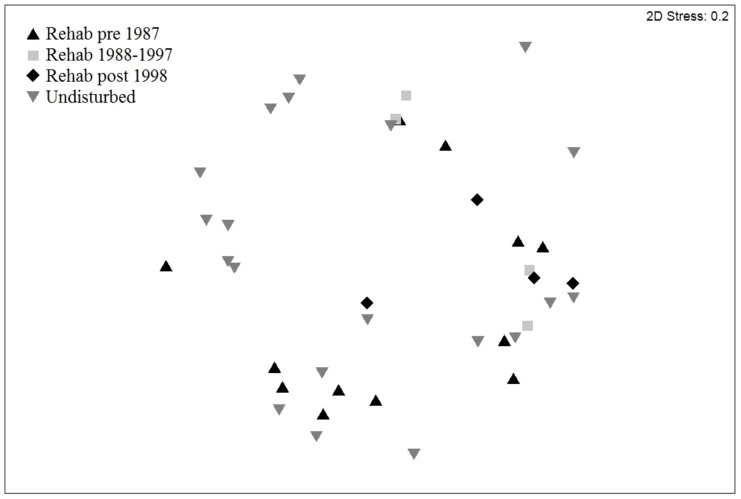
Nonparametric Multidimensional Scaling of the diet of koalas (N = 5) when their scats were found in undisturbed and rehabilitated areas.

From the leaf library, we determined that leaf cuticle characteristics were the same between trees of the same species that had grown in rehabilitated or undisturbed areas (Mann-Whitney U test statistics and p values are given in Table S1). This confirmed that we could compare leaf fragments in scats collected in rehabilitated and undisturbed areas (Table 7). Tree species found in koala scats were the same across seasons (ANOSIM, R = 0.01, p = 0.363) and individual koalas (ANOSIM, R = 0.096, p = 0.051). As the difference between individuals was almost significant, we compared, for each koala separately, the fodder trees in rehabilitated areas to those in undisturbed areas. Food trees then differed between rehabilitated and undisturbed areas (2-way crossed ANOSIM, R = 0.24, p = 0.018). An nMDS globally shows overlap between the leaf species composition from scats found in rehabilitated and in undisturbed areas although the stress level at 0.2 was at the limit of reliable representation (Figure 2).

**Table 7:** Main percentages of tree species eaten by koalas in undisturbed (N = 20 groups of scats) and rehabilitated (N = 20 groups of scats) areas.

Species		E. racemosa	E. robusta	E. resinifera	E. tereticornis	E. pilularis	E. tindaliae	E. planchon*	Lopho-stemon	Melaleuca	Angophora	Unknown
**Undisturbed**	**Mean**	**21.9**	**8.4**	**11.4**	**21.2**	**16.5**	**8.5**	**1.9**	**8.5**	**0.5**	**0.2**	**1.4**
	SD	30.6	20.9	23.4	29	30.4	14.6	8.5	7.6	1.1	0.9	2.2
	SEM	6.8	4.7	5.2	6.5	6.8	3.3	1.9	1.7	0.3	0.2	0.5
**Rehabilitated**	**Mean**	**25.2**		**9.9**	**3.8**	**30.3**	**26.1**		**3.2**		**0.2**	**1.1**
	SD	34.3		19.2	17	35.5	25.4		3.3		0.9	1.7
	SEM	7.7		4.3	3.8	7.9	5.7		0.7		0.2	0.4
**Total**	**Mean**	**23.5**	**4.2**	**10.6**	**12.5**	**23.4**	**17.3**	**1**	**5.8**	**0.3**	**0.2**	**1.3**
	SD	32.1	15.2	21.1	25.1	33.4	22.3	6	6.4	0.8	0.9	2
	SEM	5.1	2.4	3.3	4	5.3	3.5	1	1	0.1	0.1	0.3
**% of scat containing each species**		**52.5%**	**7.5%**	**22.5%**	**25.0%**	**40.0%**	**55.0%**	**2.5%**	**77.5%**	**10.0%**	**5.0%**	

* *E. planchon* is used in lieu of *E. planchon*.

**Figure 3:**
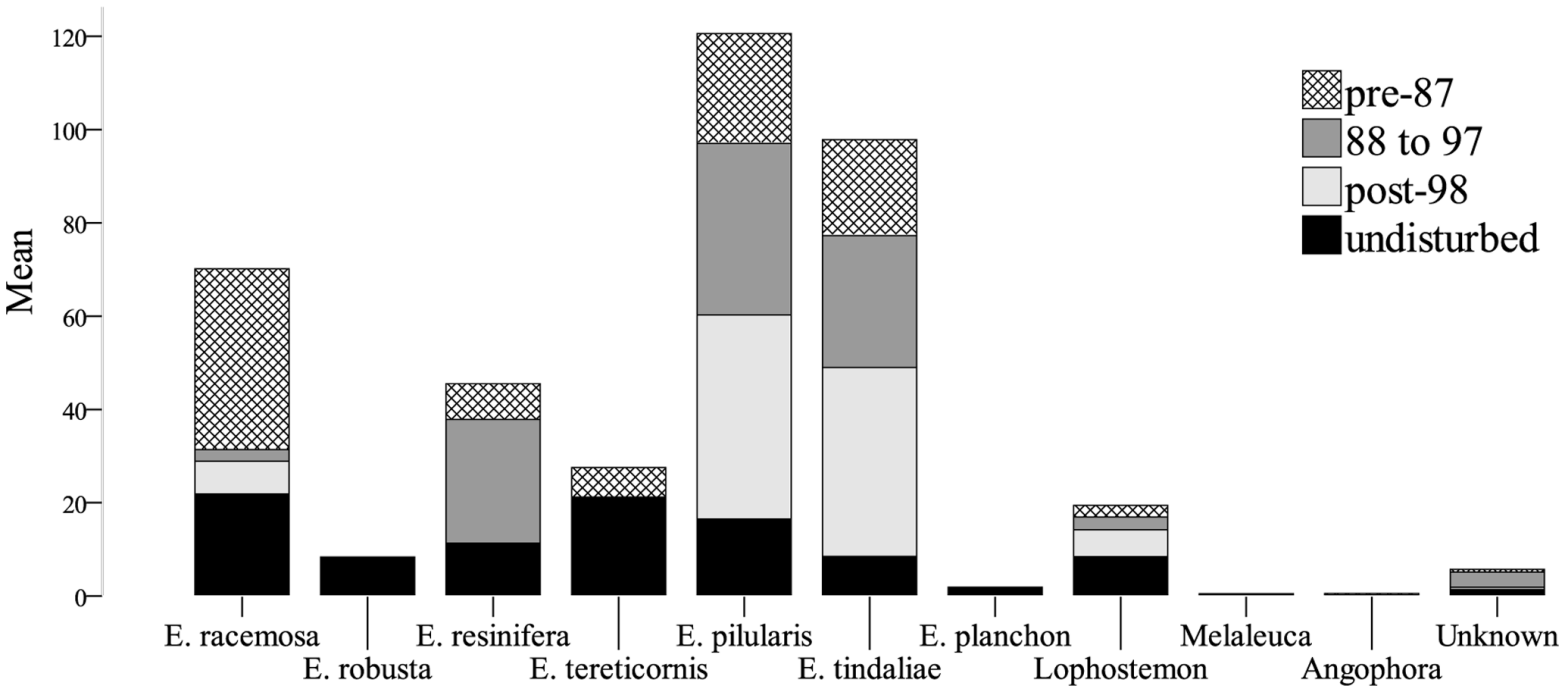
Comparison of the percentages of each tree species in koala scat groups found in undisturbed areas (N = 20) and rehabilitated areas (pre 1987 N = 12; 1988-1997 N = 4; post 1998 N = 4).

The main tree species in the scats recovered in undisturbed areas were *E. racemosa* and *E. tereticornis* (see mean percentages, SD and SEM in Table 7), with many other species well represented. In contrast, *E. pilularis*, *E. tindaliae* and *E. racemosa* were the three major species present in scats recovered in rehabilitated areas. For each of the three rehabilitated and the undisturbed areas, the percentages of each tree species in the scats were calculated (Figure 3). We compared diet evenness based on the Shannon evenness index [51], [52]. The diet in undisturbed areas was more evenly distributed across tree species than the diet in areas rehabilitated before 1997 (J_undisturbed_ = 0.84; J_pre-1987_ = 0.72; and J_88-97_ = 0.73). In turn, the diet in areas rehabilitated before 1997 was more even than the diet in post-1998 rehabilitated areas (J_post-1998_ = 0.62). However, this could reflect the difference in the number of groups of scats analysed for each area (N = 4 to N = 20): homogenised scat groups contained on average 69% of a single species and that single species was highly variable between scat groups (see SD in Table 7). Interestingly, the species most often present in scats (as opposed to present in highest quantity) was *Lophostemon* (present in 77.5% of the scat groups), followed by *E. tindaliae* (55.0%) and *E. racemosa* (52.5%, Table 7).

The density of food trees (based on the tree species found in koalas' scats) per plot in undisturbed habitats (7.5 SD = 8.6) was lower than food tree density in rehabilitated habitats (Mann-Whitney tests, pre-87: U = 94, p = 0.038; 88–97: U = 22, p = 0.011; post-98: U = 13.5, p = 0.005), whereas the food tree density for the three rehabilitated habitats (pre-87: 23.6 SD = 21.2; 88–97: 21.8 SD = 24.8; post-98: 26.1 SD = 25.1) was similar (Kruskal-Wallis test = 0.925, df = 2, p = 0.630).

### Predator index

No significant difference was found in feral predator indices between rehabilitated and undisturbed areas for any year (Mann-Whitney tests, 2003: U = 363, p = 0.061; 2005: U = 490, p = 0.736; 2009: U = 775, p = 0.673, Bonferroni's adjustment α = 0.008). To compare predator indices between years, disturbed and undisturbed areas were pooled. The predator index in 2009 was significantly higher than in 2003 and 2005 (Mann-Whitney tests, 2003: U = 1908, p = 0.001; 2005: U = 1520, p<0.001, see means, SEM and effect sizes in Table 8, Bonferroni's adjustment α = 0.008).

**Table 8:** Allen index for feral predator monitoring in 2003, 2005 and 2009.

Year	mean	SEM	effect size	
			2003	2005
**2003**	0.41	0.08		
**2005**	0.12	0.05	−0.71	
**2009**	1.22	0.19	1.98	9.17

## Discussion

Restored landscapes have the potential to create sub-optimal habitat for fauna; worse still, concerns have been raised that restoration can create ecological sinks or traps by increasing mortality, as instanced by the impact of restored road verges on butterflies [53] or mammals [54]. An interesting example of the association between restoration and ecological traps comes from the link found between particular native birds and invasive plant species. In the south-western United States, restoration projects that removed invasive plants created threats to native bird populations [55], [56]. More generally, human-modified landscapes have previously been associated with population sinks and ecological traps [57], [58], [59].

Fauna density and habitat quality are often linked, such that population sinks often have lower animal density than population sources [60], [61]. The correlation between habitat quality and fauna density, however, does not attract unanimous support, and it has been argued that density can be an indicator of habitat quality only if accompanied with data on survival and reproductive rates [7], [62]. For example, ecological traps may be characterised by lower reproductive success, lower survival rate and smaller body size and condition [63]. However, for large and long-lived mammals (such as the koala), acquisition of data to permit fitness assessment requires a substantial amount of time to collect, so in the interim it may be necessary to rely on indirect indicators to avoid irreversible impacts on recovering faunal populations. While our sample size is constrained by the longevity, relatively low natural density and slow reproductive output of koalas, information has now been gathered on koala population characteristics, in addition to the indirect measures that could influence survival rate, such as availability of food and shelter, as well as predator index.

Our first hypothesis, that rehabilitated areas could be sinks, is not supported by our observations. There was no evidence found to indicate that rehabilitated habitat was of low quality (in terms of tree species richness and density, or predator density) nor that koalas were present in lower density, were in poorer condition, or had lower reproductive output in rehabilitated areas. A similar density of scats was found in rehabilitated and undisturbed habitats. Moreover, in our study, the radio-tracked koalas spent equivalent time in rehabilitated and undisturbed areas. These koalas did not appear to be old, sick or dispersing animals that may have been evicted from primary habitat. Instead, koalas of various ages, in good general and breeding condition were observed using rehabilitated areas. The majority of females using rehabilitated areas were carrying young (5/6), and the one koala located only in rehabilitated areas carried two back young successively during the study. As the radio-tracking of koalas occurred on a weekly basis, it could be argued that short excursions of this koala out of rehabilitated areas could be missed. Thus this female koala found 100% in rehabilitated areas on the basis of VHF data, was fitted with a GPS collar (one position recorded every four hours) and the GPS data confirmed that she did not leave rehabilitated areas, even for short excursions into the undisturbed areas [64]. With the caveat that our sample size is not large, there is nothing in our data to suggest that rehabilitated areas are acting as population sinks, but rather that koalas select rehabilitated areas as a substantial part of their home ranges.

On the basis of vegetation characteristics, it is not surprising that rehabilitated areas could indeed be as attractive to koalas as undisturbed areas, if not even more attractive. All rehabilitated habitats used by koalas had higher tree density and species richness, as well as a similar canopy cover (for all habitats rehabilitated before 1997) than undisturbed habitats, along with a higher density of food trees than undisturbed habitats. In particular, the habitats rehabilitated after 1998 contained young, fast growing trees, which could enhance leaf quality for koalas [20], [21]. Thus, the rehabilitated habitats possessed characteristics likely to make them attractive koala habitats and this was evidenced by their use by koalas. This fauna recolonisation outcome was the desired goal and the motivation for undertaking the progressive improvements in rehabilitation methodology.

Nonetheless, it could have been that the cues that attracted koalas to the rehabilitated areas were disconnected from the net value of rehabilitated habitats for the species in terms of reproductive or survival rates, thus opening the potentiality for an ecological trap [58], [65]. A well-documented example of misleading cues involves mayflies and dragonflies, for which crude oil surfaces and asphalt roads were found to be more visually attractive than water surfaces, thereby diverting them from their breeding sites [66], [67], [68]. However, our observations do not indicate that the reproductive output of koalas spending time (up to 100%) in rehabilitated areas is lower than in undisturbed areas.

We assessed the likelihood of our second hypothesis, the creation of an ecological trap, on the basis of study findings on other processes able to create ecological traps. Ecological traps that provide lesser foraging quality and/or quantity can have dramatic consequences on fauna fitness, like slower growth rates and smaller adult size [63] or even starvation [69]. As koalas depend on a low-nutrient diet [70], readily accessible food is crucial to keep the energy ratio of food intake/travel cost in a viable range [71]. In this study, food quantity and diversity are unlikely to limit habitat suitability, as native tree density and species richness, as well as food tree density, were higher in rehabilitated than undisturbed habitats. Diet in rehabilitated and undisturbed areas was also similar; however, koalas may rely on fewer food species in rehabilitated than in undisturbed areas and this warrants further consideration.

The number of tree species eaten by a folivore can be critical and may reflect the physiological constraints imposed on the individual [72]. Indeed, different eucalypt species can have different chemical defences, or toxins [73] and the food intake of some folivores may be limited by these toxins [72]. Some folivores are known to be able to increase food consumption by switching between species with different toxins, which in turn use different detoxification pathways [74]; however, evidence for the extent to which plant foliar toxins play a major role in dietary selection by koalas is equivocal [75]. Thus if the lower number of species eaten in some rehabilitated compared to undisturbed areas on NSI is found to be a consistent phenomenon, it would be useful to investigate if this can be attributed to trees in rehabilitated areas possessing a lower level of toxins which could thus enable a larger quantity of the same species to be eaten, or not. Dietary intake by koalas has been shown to depend on water and nutrient (such as nitrogen) content [76], [77], [78], [79] and it has also been proposed that foliar nutrients and toxins can be influenced by soil characteristics [80], [81]. Worthwhile future investigations could compare the foliage quality in rehabilitated areas, where the soil has been disturbed, with foliar composition in undisturbed areas. This also presents an opportunity to investigate further the influence of soil fertility and leaf chemistry on koala dietary selection.

Shelter is important to protect animals from predators and against the elements. In particular, thermoregulatory constraints could influence the selection of roosting trees by koalas [71], [82]. Ecological traps have been known to result from improper or disrupted shelter from predators [83] or from inappropriate temperature [84], [85]. In our study, rehabilitated areas seemed to provide suitable shelter for koalas as indicated by similar rates of re-using the same roosting trees in rehabilitated areas as in undisturbed areas. Koalas also used the same number of roosting species in rehabilitated and undisturbed areas. A potential problem could come from the size of roosting trees used by koalas: they were smaller in rehabilitated than in undisturbed areas, but other studies demonstrate that elsewhere koalas also use small trees [86].

The consequence of ecological traps most frequently reported in the literature is increased predation (e.g. [87], [88], [89], [90]). In particular, anthropogenically disturbed landscapes have created ecological traps by increasing predation on birds [63], [91] and lizards [83], [92]. From our results on predator presence in the area, it appears that predator density is not higher in rehabilitated than in undisturbed areas. However, the general increase of predator index across the years in both rehabilitated and undisturbed areas of NSI is of concern. Further research should also focus on whether terrestrial predator movements are facilitated in rehabilitated areas. For instance, rehabilitated areas could be associated with increased track density and/or bush penetrability (possibly via a less complex ground layer). Moreover, predation risk for koalas increases with the amount of time spent on the ground [93], which might be expected to be associated with fodder and roost tree densities.

In the rehabilitated habitat on NSI, trees were found to be smaller, which could imply that to gain access to the same quantity of foliage, koalas might have to change trees more often than in undisturbed habitats. However, since koalas prefer to feed on tip growth [94] and the proportion of tip growth tends to be greater (and the tips more accessible) in younger trees, koalas may be able to meet their browse intake more easily in the smaller trees, even though the larger trees in the undisturbed areas have bigger canopies with a higher foliar mass (but proportionately less tip growth). Also, trees are closer together in the rehabilitated areas, meaning that koalas are likely to have less distance to travel from one tree to the next. Ongoing research is currently attempting to quantify in more detail what impact, if any, living in rehabilitated areas has on koala movement patterns [64] and any relationship this might have with the potentially significant threat posed by predation.

Though based on relatively small numbers of experimental animals, the results on population characteristics, availability of food and shelter, as well as predator index, indicate that our third hypothesis has best support: rehabilitation of previously mined areas seems to provide suitable new koala habitat. We found no evidence that the creation of a population sink or an ecological trap is likely. Research on predation risk and long-term survival rates of koalas would be a valuable addition to confirm this conclusion.

Limitations of our study make this conclusion a preliminary one. Due to the amount of time necessary to gather the information presented above, only small sample sizes could be included (i.e. the number of koalas and the number of scats for dietary analysis). Another limitation for determining the creation of population sinks or ecological traps is that we were unable to establish population demographics (e.g. survival rate) directly and we had limited opportunity to determine differential reproductive output. Finally, for an ecological trap to be appropriately assessed, data on animal behaviour are necessary (i.e. to show that individual koalas chose rehabilitated areas instead of available undisturbed areas). To study habitat selection by animals, choice experiments or settlement patterns in migratory species [7] are often employed, but are difficult to implement for koalas. Comparison of habitat availability with habitat use is also complicated for this species; the diversity and heterogeneity of koala habitats and the relative paucity of reports of well-designed population ecology and behavioural studies in the field conspire to preclude a substantial foundation for detailed habitat choice studies by koalas. There is no well-founded evidence based consensus on key elements of koala habitat quality, let alone selection. The limitations can be resolved with longitudinal studies, but survival and reproductive data are always difficult to gather for long-lived and slow-breeding species.

Interestingly, our results tend to indicate that koalas could be more adaptable than previously speculated [22], [95]. Notably, koalas were observed to change their habits to occupy newly available habitat. Indeed, koalas were found using rehabilitated habitats established from 6 to 31 years previously. These rehabilitated habitats differed from undisturbed koala habitat in structure (e.g. tree size, bare ground, canopy) and species composition from undisturbed koala habitat. Moreover, radio-tracked koalas roosted in and ate a different suite of tree species in undisturbed and rehabilitated areas. These comparisons of behaviour appear to be reliable, since they were performed for the same individual in both habitats, thus controlling for bias resulting from differences in individual preference. The relative adaptability of koalas to disturbances that we observed on NSI mirrors results found in logging areas [18], [96], agricultural [101] and fragmented landscapes [93], [97], [98]. Some forms of disturbed areas thus can still retain conservation value for koalas. This supports a non-Manichean view of the landscape matrix, where a landscape can contain many shades of disturbance intensity and habitat suitability [99].

## Conclusion

The results of this study are promising for the restoration of previously mined areas on NSI, and support previous evidence that when commitment to rehabilitation is strong enough, suitable habitat for fauna can be created, even after the extreme disturbance produced by mining. The success achieved so far, demonstrates that using benchmarking and an adaptive management approach to improve rehabilitation is worth the effort. We hope that this will encourage more mining companies and other industries to follow this example and that these findings will result in setting the benchmark for a new legislative framework that includes fauna in assessment criteria of rehabilitation success.

As human beings try to salvage some of the ever increasing areas of land disturbed by their activities, the challenge is not only to put in the substantial effort required to restore fauna habitat attributes, but also to ensure that these areas will support a population with similar reproductive and survival rates to those in comparable undisturbed original habitat. This will ensure restoration results in maintaining wildlife populations and not the reverse [91]. More research is needed to determine if success is possible in the “acid test” of applying our ecological understanding of the world [100] and the extent to which nature can be restored. In the meantime, it must be emphasised that though habitat restoration is important, it is no substitute for the protection of adequate amounts of undisturbed environment [2], [101], [102].

## Supporting Information

Figure S1 **Boxplots of the number of scats and selected vegetation characteristics of plots in rehabilitated (classified by method) and undisturbed koala habitats (dashed line represents undisturbed value).**
(DOCX)

Table S1 **Results of Mann-Whitney U tests comparing stomatal lengths for some of the NSI tree species in the leaf library between trees sampled in rehabilitated areas and trees sampled in undisturbed areas.**
(DOCX)
