# Correction: Potential 'Ecological Traps' of Restored Landscapes: Koalas *Phascolarctos cinereus* Re-Occupy a Rehabilitated Mine Site

**DOI:** 10.1371/journal.pone.0130115

**Published:** 2015-06-09

**Authors:** Romane H. Cristescu, Peter B. Banks, Frank N. Carrick, Céline Frère, The PLOS ONE Staff

The authors and *PLOS ONE* staff wish to amend the Competing Interests Statement for Dr. Romane Cristescu for this article,[1] which should have included additional information in relation to potential competing interests relevant to this work. The authors and *PLOS ONE* staff regret that a declaration of this competing interest was removed in error during the submission process, and would like to add this disclosure to the declaration:

Dr. Cristescu has been an employee of Sibelco Australia—Mineral Sand since July 2011. During the course of the study (2008–2010), Dr. Cristescu completed casual work on North Stradbroke Island, including sporadic administrative tasks unrelated to this study for Consolidated Rutile Limited (CRL), the company subsequently taken over by Sibelco.

Though there was no direct funding of the project as originally declared, Sibelco Australia (and CRL beforehand) provided indirect support. The Centre for Mined Land Rehabilitation previously received research funding from the company, mainly for vegetation work but also for some previous Koala research; the project used some resources (e.g. radio-tracking transmitters and receivers) procured for these earlier studies. Likewise, The University of Queensland received funding for the Moreton Bay Research Station at Dunwich. This support did not affect the design, conduct or conclusions of the research.
