# Supplementary material for: Potential ‘Ecological Traps’ of Restored Landscapes: Koalas Phascolarctos cinereus Re-Occupy a Rehabilitated Mine Site
Source: PLoS One. 2013 Nov 25;8(11):e80469. doi: 10.1371/journal.pone.0080469 (PMC3839991; doi:10.1371/journal.pone.0080469)
Supplement: Figure S1 — Boxplots of the number of scats and selected vegetation characteristics of plots in rehabilitated (classified by method) and undisturbed koala habitats (dashed line represents undisturbed value). (DOCX) [file pone.0080469.s001.docx]

**S1: Boxplots of the number of scats and selected vegetation characteristics of plots in rehabilitated (classified by method) and undisturbed koala habitats (dashed line represent undisturbed value**)
