# Supplementary material for: Potential ‘Ecological Traps’ of Restored Landscapes: Koalas Phascolarctos cinereus Re-Occupy a Rehabilitated Mine Site
Source: PLoS One. 2013 Nov 25;8(11):e80469. doi: 10.1371/journal.pone.0080469 (PMC3839991; doi:10.1371/journal.pone.0080469)
Supplement: Table S1 — Results of Mann-Whitney U tests comparing stomatal lengths for some of the NSI tree species in the leaf library between trees sampled in rehabilitated areas and trees sampled in undisturbed areas. (DOCX) [file pone.0080469.s002.docx]

**Table S1: Results of Mann-Whitney U tests comparing stomata lengths for some of the NSI tree species in the leaf library between trees sampled in rehabilitated areas and trees sampled in undisturbed areas**
